# Effects of warming seasonal rotational grazing on plant communities' structure and diversity in desert steppe

**DOI:** 10.1002/ece3.9748

**Published:** 2023-01-19

**Authors:** Wen Li, Tingting Su, Yan Shen, Hongbin Ma, Yao Zhou, Qi Lu, Guohui Wang, Zhuo Liu, Jianping Li

**Affiliations:** ^1^ Key Laboratory for Model Innovation in Forage Production Efficiency Ministry of Agriculture and Rural Affairs, P.R. China Ningxia University Yinchuan China; ^2^ Ningxia Grassland and Animal Husbandry Engineering Technology Research Center Ningxia University Yinchuan China; ^3^ School of Agriculture Ningxia University Yinchuan China; ^4^ Breeding Base for State Key Laboratory of Land Degradation and Ecological Restoration of Northwest China Ningxia University Yinchuan China

**Keywords:** desert steppe, functional groups, plant community characteristics, species diversity, warming seasonal rotational grazing

## Abstract

Grazing is the basic way of grassland utilization, and reasonable grazing is an important way to maintain the health of the grassland ecosystem. However, the traditional grazing time in warming seasons is negative for sustainable desert steppe ecosystem. Determining reasonable grassland grazing methods is to remain a critical issue for the ecological conservation and rational utilization of desert steppe. Therefore, our objectives were to explore the effects of warming seasonal rotation grazing on the species diversity and functional diversity of grassland plants and to reveal controlling factors of plant community diversity. The warm‐season rotational grazing modes included traditional time of grazing (FG), delayed start of grazing (YG), early end of grazing (TG), delayed start early end of grazing (YT), and enclosed steppe (CK). The results showed that the important value of *Agropyron mongolicum* of the gramineae and *Lespedeza potaninii* of the leguminosae in YG increased by 12.10%–120.66% and 23.57%–34.25% than other treatments (CK, FG, TG, and YT), respectively. Therefore, the YG treatment has more advantages on the IV of *A. mongolicum* of the gramineae and *L. potaninii* of the leguminosae. Warming seasonal rotational grazing (FG, YG, TG, and YT) significantly increased the important value of *Leymus secalinus* by 51.43%–79.64% compared with CK (*p* < .05). Compared with CK, FG and YG promoted the growth of gramineae and appropriately reduced the proportion of forbs. There was no significant difference in the Shannon–Wiener index between grazing treatments and CK, while the Shannon–Wiener index in YT increased by 21.43% and 15.71% compared with FG and YG (*p* < .05). The functional richness index in FG and YG significantly decreased by 19.05%–23.81% compared with CK and TG (*p* < .05). The results of the redundancy analysis showed that the diversity of plant communities was mainly affected by soil‐available nitrogen. These observations indicated that delayed start of grazing can improve the diversity of plant communities by increasing the important value of dominant plants in the community and promoting the growth of gramineous and leguminous plants, thereby optimizing the composition of community structure. Our findings can provide a theoretical basis for formulating a reasonable and scientific grazing period in desert steppe.

## Graphical abstract

Grazing is the basic way of grassland utilization and an important way to maintain the health and production stability of grassland ecosystem. However, the traditional grazing in winter and spring is negative for the sustainable development and management of ecosystem in desert steppe. Determining reasonable grassland grazing methods is to remain a critical issue for the ecological conservation and rational utilization of desert steppe. Therefore, our objectives were to explore the effects of warming seasonal rotation grazing on the species diversity and functional diversity of grassland plants, and revealing controlling factors of plant communities. The results of the redundancy analysis showed that the diversity of plant communities was mainly affected by soil‐available nitrogen. These observations indicated that delayed start grazing can improve vegetation diversity. Our findings can provide a theoretical basis for formulating a reasonable and scientific grazing system in desert steppe.

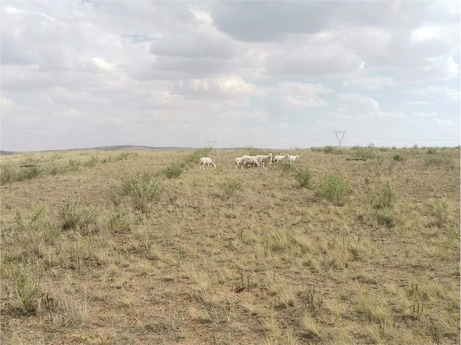

## 1 INTRODUCTION

Grassland ecosystem is the largest terrestrial ecosystem and an essential component of the global natural ecosystems (Tian et al., 2020). Grassland plays a vital role in climate regulation, nutrient cycling, and soil and water conservation (Chadaeva et al., 2021; Villoslada Peciña et al., 2019). Moreover, natural grassland is also the critical material support for developing grassland animal husbandry (Amidzic et al., 2020). Therefore, grazing is the primary disturbance factor in natural steppe systems. Livestock has essential effects on the structure and function of plant communities through feeding, trampling, and excretion (Benjamin et al., 2014; Schönbach et al., 2011; Shan et al., 2011), and even affects the succession process and direction of communities (Guo et al., 2021). However, long‐term and unreasonable grazing methods will directly change the morphological structure, productivity, and grass seed structure of the steppe (Song et al., 2020; Zhang, Wang, et al., 2018) and then affected the soil structure and nutrient cycle of the steppe and the production performance of livestock (Boval & Dixon, 2012), resulting in significant reduction in steppe regeneration capacity, biomass and nutrient content, which eventually led to steppe degradation (Peco et al., 2017). Therefore, the utilization of the grassland in a reasonable way of grazing is a hot topic, and it has received more and more attention from scholars.

Currently, many researchers focused on how to use grassland resources with reasonable grazing methods, including the impact of different grazing intensities and different grazing methods on grassland productivity, species composition, vegetation diversity, and soil properties. Research showed that aboveground biomass is often increased by intermediate grazing compared to that by light and heavy grazing, due to the fact that medium grazing resulted in a higher species diversity than no or low grazing (Gong et al., 2014; Yan et al., 2013). Grazing intensity can change plant community structure, soil microenvironment, and soil microbial diversity and activity (Stavi et al., 2008). Wang et al. (2019) reported that compared with traditional free grazing, rotational grazing can significantly improve the productivity, stability, resilience, and other system functions of the grassland ecosystem; the standing stock and productivity of dominant species have been improved. Under moderate grazing intensity, warm‐season grazing increased forb functional group proportion, plant density, and evenness index, and it was more conducive to the maintenance of species diversity and evenness of steppe community compared with cold‐season grazing (Wu et al., 2017). However, cold‐season grazing was conducive to the accumulation of aboveground biomass of plant community in desert steppe (Tian et al., 2020). Cox et al. (1989) considered that the best grazing practices were to graze in spring but not in autumn as green grass is of the best quality in spring, while yields and quality decline rapidly due to dried forage in autumn. Wu et al. (2017) also found that both summer and winter grazing reduced the proportion of gramineous plant functional groups and increased the proportion of forbs plant functional groups. However, cold‐season grazing was conducive to the accumulation of aboveground biomass in steppe community (Tian et al., 2020). Nie and Zollinger (2012) reported that compared with continuous grazing, delayed grazing in spring and autumn increased the tiller density of perennial grass, moreover, the ground coverage increased by 27% on average after delayed grazing in autumn. In general, different grazing regimes had different effects on plant composition. Seasonal grazing provided a specific period for plant recovery in grassland, which might increase resistance to grazing and alter the development of plant composition, especially in heavy grazing (Sternberg et al., 2015). Wu et al. (2017) showed that seasonal grazing mainly affected soil bulk density, water content, and distribution of soil carbon, nitrogen, phosphorus, and other nutrients through livestock activities, and ultimately affected plant community composition and species diversity. Therefore, grazing regimes (seasonal grazing) are essential for grassland management. Recent interests in the effects of grazing on plant functional traits and functional diversity, however, further research is needed to explore the relationships between warming seasonal grazing and plant community composition and functional groups, and environmental factors controlling vegetation diversity under different warming seasonal grazing regimes.

Chinese desert steppe covers an area of 1.73 × 10^8^ ha, accounting for 22.70% of the national territory, and plays essential roles in livestock production and ecosystem protection (Xu et al., 2019). The area is a typical semi‐arid region with a drought climate and is also a fragile habitat for animals and plants. Therefore, the sustainable utilization and management of desert steppe is always an issue of concern to scholars. Previously reported that rational grazing of grassland was beneficial to the stability of grassland vegetation community, the increase in species diversity, and the maintenance of the multifunction of the grassland ecosystem. However, the effects of warming seasonal rotational grazing on the composition and diversity of plant community in desert steppe need to be further explored. Therefore, in our study four warming seasonal rotational grazing methods were conducted in desert steppe, including the enclosed steppe (CK), traditional time of grazing (FG), early end of grazing (TG), delayed start of grazing (YG), and delayed start early end of grazing (YT). The main objectives of our study were to: (1) explore the impacts of different grazing times on the composition of functional groups of plant communities, (2) responses of the diversity of plant communities to different grazing times, and (3) reveal the relationships between plant community diversity and soil environmental factors.

## 2 MATERIALS AND METHODS

### 2.1 Study area

**FIGURE 1:**
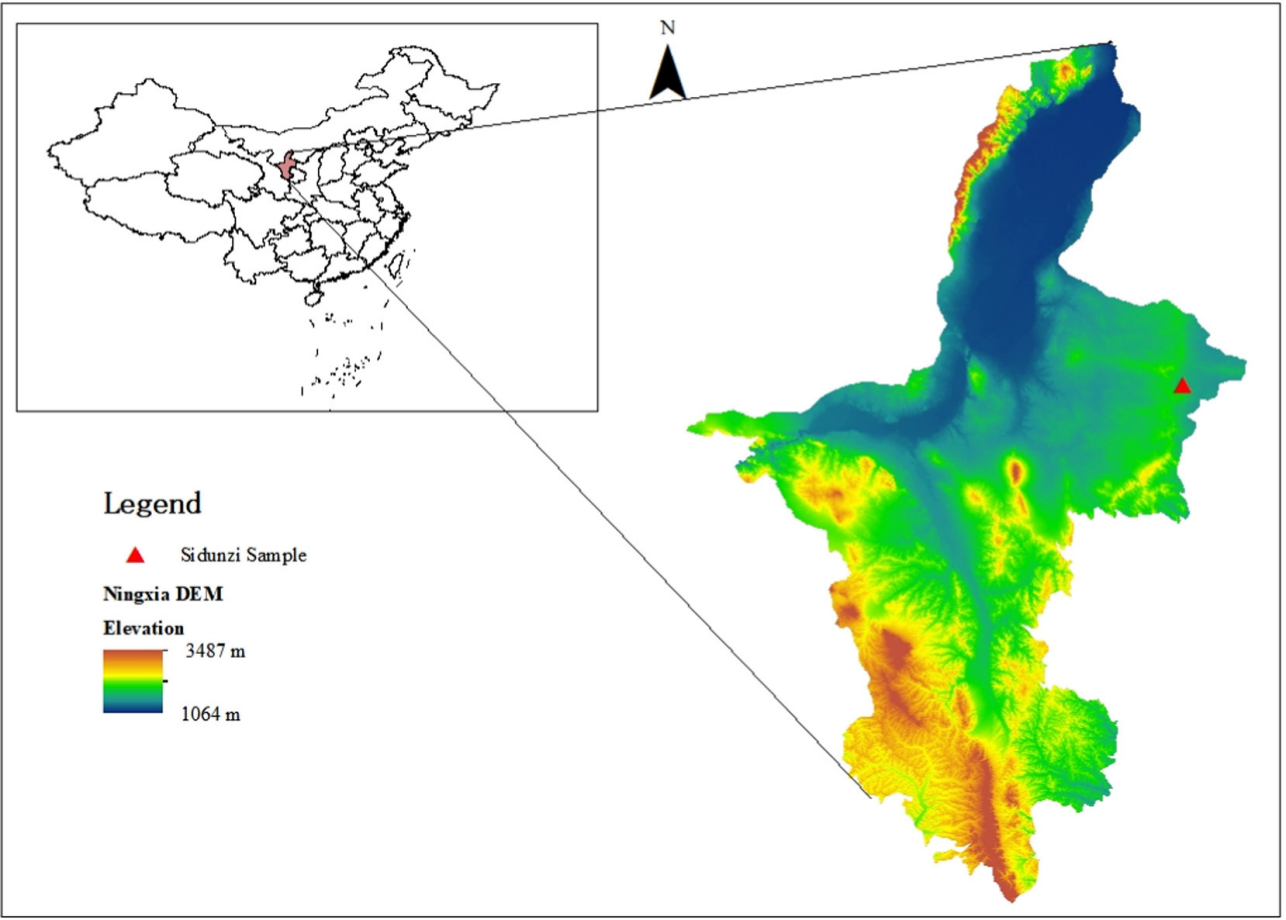
Location of study area

The study was conducted in the Sidunzi village, Yanchi County, Ningxia Hui Autonomous Region, China (107°16′27″ E, 37°45′24″ N, altitude 1450 m), which is desert steppe and near the south edge of the Mu Us Desert. Since 2003, this area has been closed to grazing because of vegetation degradation in desert steppe. It is an essential farming‐pastoral zone with a temperate continental climate in northern China. Mean annual precipitation is 292.30 mm, and approximately 72.80% occurred between June and September (Figure 1), with a frost‐free period of about 162 d. The annual evaporation is about 2136 mm, the average temperature is 7.7°C, and the cumulative thermal time (≥0°C) is 3430.3°C. The soil type is alkaline sierozem with a particle distribution of 11.2%–14.3% clay, 30.1%–44.5% silt, and 45.4%–50.9% sand. The average content of Soil organic carbon (SOC) is 4.34 g·kg^−1^, total nitrogen content (TN) is 0.24 g·kg^−1^, and total phosphorus content (TP) is 0.23 g·kg^−1^. The natural vegetation is dominated by *Agropyron mongolicum*, *Lespedeza potaninii*, and *Stipa breviflora*, followed by sparse species such as *Polygala tenuifolia* and *Astragalus melilotoides*.

### 2.2 Experimental design

**FIGURE 2:**
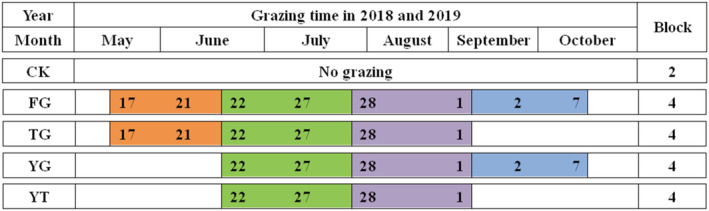
Specific time of rotational grazing treatment. CK—enclosed steppe; FG—traditional time of grazing; TG—early end of grazing; YG—delayed start of grazing; YT—delayed start and early end of grazing. Different colors represent different rotational grazing cycles.

The grazing experiment was conducted in a 15a enclosed desert steppe from May to October in 2018 and 2019. It involved five treatments with a completely randomized design, including traditional time of grazing (FG), delayed start of grazing (YG), early end of grazing (TG), delayed start and early end of grazing (YT), and the control enclosed grassland (CK—enclosed steppe). In total, 18 plots were sampled, covering an area of 4.27 ha for each. The grazing intensity was determined to be 0.75 sheep·units ha^−2^, and the grazing mode was rotation grazing in four areas (Wang et al., 2019; Yang et al., 2006). The grazing period is 36 d. In each rotation grazing period, the grazing days of the plot are 9 d and the grazing season is 144 d. In addition, electric fences were set among the treated areas to prevent sheep from running out. Thirteen Tan sheep with similar weight (about 45 kg) was chosen. Detailed information on the grazing time and rotation cycle is shown in Figure 2.

### 2.3 Measurements of plant diversity and soil properties

The vegetation community characteristics and soil properties under different treatments were investigated in September 2019. Ten subplots (1 m × 1 m for each) were randomly conducted in each treated plot. The species of each subplot were recorded during the monitoring period. Plant coverage, height, frequency, and density were also recorded in each subplot (Shen et al., 2021; Zhou et al., 2020). And all species were divided into three classes (gramineous grasses, leguminous grasses, forbs grasses) into the subplots, and then collected and dried at 65°C to measure aboveground biomass. It mainly included *Cynanchum komarovii*, *Agropyron mongolicum*, *Stipa breviflora*, *Leymus secalinu*, *Astragalus melilotoides*, *Polygala tenuifolia*, *Scorzonera austriaca*, *Convolvulus ammannii*, *Salsola collina*, *Artemisia scoparia*, etc.

Soil samples were collected without plant litter and roots in the soil layers of 0–10 cm, 10–20 cm, 20–30 cm, and 30–40 cm in the subplot by multi‐point mixing method. The chemical and physical characteristics of soil samples were measured, including available nitrogen (AN), soil porosity (SP), soil moisture content (SMC), total nitrogen (TN), available potassium (AK), and organic carbon (OC). Detailed measurements of soil chemical and physical properties were reported by He et al. (2021) and Wu et al. (2010).

The data in the same subplot were pooled and merged to calculate species' important value, species diversity (Patrick richness, *R*; Shannon–Wiener diversity index, *H*; Pielou evenness index, *J*; and Simpson dominance index, *D*), functional richness (*FRic*), functional evenness (*FEve*), functional dispersion (*FDiv*). The calculation formula for each indicator is as follows:

1. Important value (IV)

(1) $$\mathrm{IV}=\frac{\mathrm{RC}+\mathrm{RH}+\mathrm{RB}}{3},$$

where *RC* is relative coverage; *RH* is relative height, and *RB* is relative biomass.

2 Species diversity

Four commonly used species diversity indexes were selected in this study, namely *R*, *H*, *J*, and *D*. The calculation formula is as follows (Jäschke et al., 2020):

(2) R=S

(3) $$P_{i}=n_{i}/N$$

(4) $$H=-\sum_{i=1}^{S}P_{i}\ln P_{i}$$

(5) $$J=H^{\prime }/\ln S$$

(6) $$D=1-\sum_{i=1}^{S}P_{i}^{2},$$

where *S* is the number of species, *P*_*i*_ is the proportion of individuals of species *i* in the quadrat, *n*_*i*_ is the biomass of species *i* in the quadrat, and *N* is the total biomass of all species in the quadratic.

3 Functional richness

It is obtained by calculating the area or volume of the smallest polygon in the trait space, expressed as a functional richness index (*FRic*), which is used to measure the size of the ecological niche occupied by species, reflecting the stability and anti‐disturbance ability of the community (Mason et al., 2005).

(7) FRic=SFic/Rc,

where *SFic* is the niche space filled by the species within the community, and *Rc* is the absolute range of the character (Mason et al., 2005).

4 Functional evenness

The functional evenness index (*FEve*) is a measure of the distribution law of species' functional traits in the occupied trait space and is used to predict resource utilization (Song et al., 2011). Its calculation formula is:

(8) $$\text{FEve}=\frac{\sum_{l=1}^{S-1}\min\left({\mathrm{PEW}}_{l}-\frac{1}{S-1}\right)-\frac{1}{S-1}}{1-\frac{1}{S-1}}$$

where *S* is the number of species, *PEW*_*l*_ is the weight of branch length, *EW*_*l*_ is the branch length, *l* is the branch length, and *W*_*i*_ and *W*_*j*_ are the relative abundances of species, respectively.

5 Functional divergence

The multidimensional functional divergence is expressed by the functional divergence index (*FDiv*), which measures the maximum divergence of the abundance distribution of community functional traits in the trait space. It is used to predict the dispersal of resources (Jäschke et al., 2020). The specific calculation formula is as follows:

(9) $$\text{FDiv}=\frac{\Delta d+\bar{\mathrm{dG}}}{\Delta \left|d\right|+\bar{\mathrm{dG}}},$$

where Δ*d* is the dispersion degree weighted by abundance, and $\bar{\mathrm{dG}}$ is the average distance between species *i* and the center of gravity.

### 2.4 Statistical analysis

Descriptive statistics were conducted for the plant diversity index (*R*, *H*, *J*, *D*, *FRic*, *FEve*, *FDiv*). Differences in essential values, species diversity index, and functional diversity index among different treatments were identified by the method of analysis of variance (ANOVA) by the SPSS 21.0 software. Canoco5.0 was used for the redundant analysis of plant species diversity, biomass, and soil indicators. Origin Pro 2022 software for Windows was used to draw the figures and perform the statistical analyses.

## 3 RESULTS

### 3.1 Species composition and importance value

The steppe vegetation composition was dominated by perennial plants under the different warm‐seasonal rotation grazing treatments (Table 1). Under short‐term seasonal rotational grazing, the dominant plants of desert steppe mainly included *A. mongolicum*, *S. breviflora* of the gramineae and *L. potaninii* of the leguminosae, etc. The IV (important value) of *A. mongolicum* of the gramineae and *L. potaninii* of the leguminosae in YG were highest, and significantly increased by 12.10%–120.66% and 23.57%–34.25% than other treatments (CK, FG, TG, and YT), respectively. Warming seasonal rotational grazing (FG, YG, TG, YT) significantly increased the IV of *L. secalinus* of the gramineae by 51.43%–79.64% compared with CK (*p* < .05). The IV of *P. tenuifolia* and *S. collina* of the forbs in FG was lowest and significantly decreased by 28.30%–35.57% and 48.93%–74.80% than others (TG, YG, YT, and CK) (*p* < .05), respectively. However, no significant difference was observed in *S. breviflora* among YG, CK, and FG, and the same trend occurred in *C. komarovii* of the forbs among different treatments (*p* > .05). Furthermore, the decreasing patterns were observed in FG and YG for *S. austriaca* in composite and *S. collina* in chenopodiaceae comparing with CK (*p* < .05). Therefore, the YG treatment has more advantages on the IV of *A. mongolicum* of the gramineae and *L. potaninii* of the leguminosae.

**TABLE 1:** Important values of main species in dessert steppe under warm‐seasonal rotation grazing treatments

Functional group	Species	Important value (%)				
		CK	FG	TG	YG	YT
Gramineae	*A. mongolicum*	8.70 ± 0.03b	7.60 ± 0.01cb	14.96 ± 0.03a	16.77 ± 0.01a	10.37 ± 0.01b
	*S. breviflora*	9.05 ± 0.01a	9.76 ± 0.02a	7.80 ± 0.02b	9.42 ± 0.01a	7.84 ± 0.01b
	*L. secalinu*	2.80 ± 0.04c	4.24 ± 0.01ab	5.03 ± 0.02a	4.51 ± 0.01ab	4.61 ± 0.03ab
Leguminosae	*L. potaninii*	10.12 ± 0.02b	9.90 ± 0.01b	10.69 ± 0.02b	13.21 ± 0.01a	9.84 ± 0.03b
	*A. melilotoides*	10.39 ± 0.03a	2.79 ± 0.01c	5.10 ± 0.02b	3.35 ± 0.01c	5.35 ± 0.04b
Forbs	*C. komarovii*	4.00 ± 0.01a	5.67 ± 0.01a	3.87 ± 0.01a	4.59 ± 0.03a	4.92 ± 0.01a
	*P. tenuifolia*	6.41 ± 0.02a	4.13 ± 0.01b	6.38 ± 0.03a	5.98 ± 0.01a	5.76 ± 0.01a
	*S. austriaca*	5.69 ± 0.01a	1.83 ± 0.01c	4.29 ± 0.01ab	2.18 ± 0.01c	4.29 ± 0.02ab
	*C. ammannii*	6.00 ± 0.01a	3.11 ± 0.03bc	2.44 ± 0.02c	4.60 ± 0.03b	2.62 ± 0.02c
	*S. collina*	7.58 ± 0.03a	1.91 ± 0.01c	6.93 ± 0.01a	3.74 ± 0.02b	4.95 ± 0.04b
	*A. scoparia*	1.88 ± 0.03b	10.54 ± 0.01a	4.45 ± 0.02b	3.73 ± 0.04b	3.01 ± 0.02b

*Note*: CK—enclosed steppe; FG—traditional time of grazing; TG—early end of grazing; YG—delayed start of grazing; YT—start late and end early of grazing. Different small letters indicated significant differences among different treatments at *p* < .05.

**FIGURE 3:**
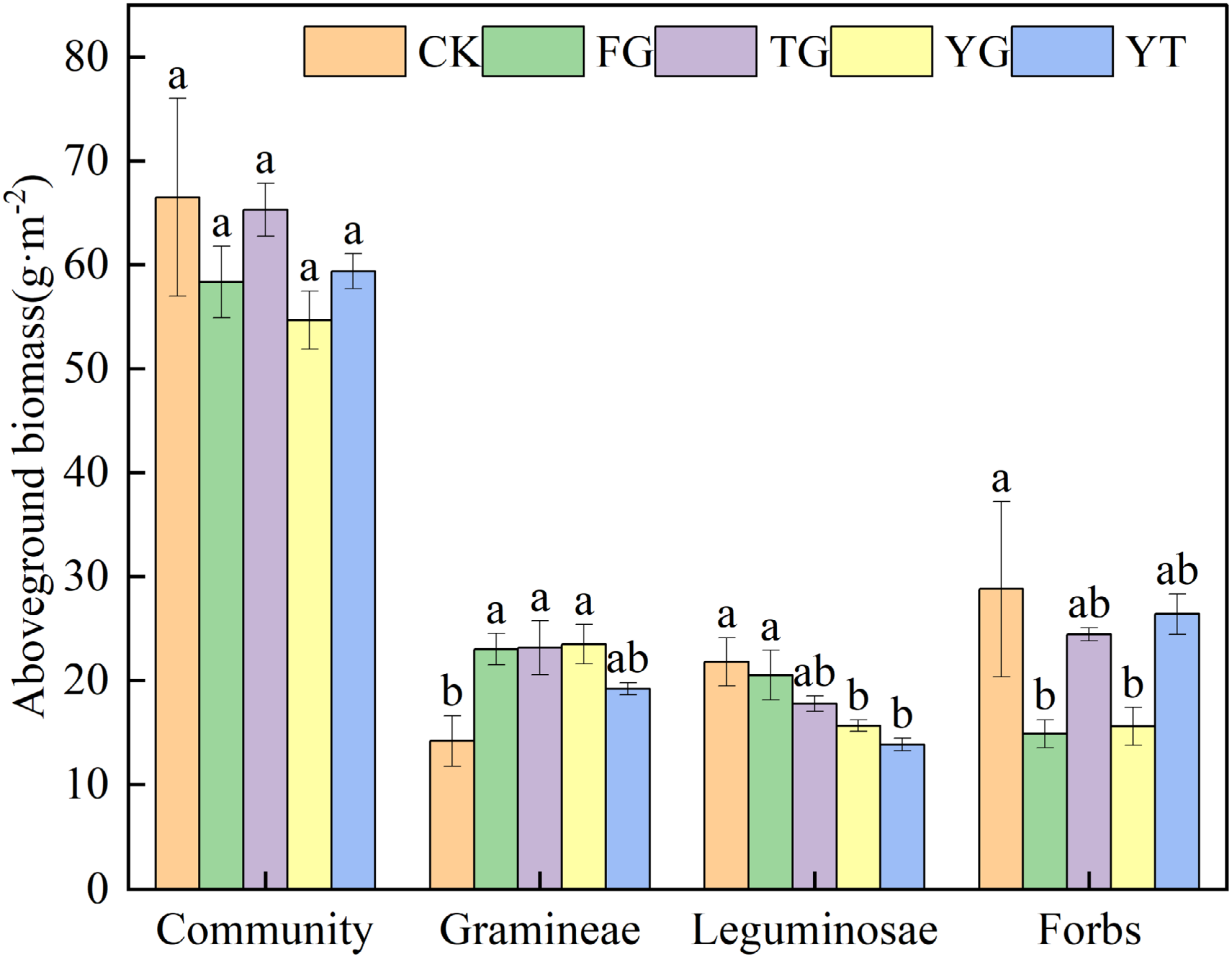
Mean aboveground biomass of different vegetation types under different warming rotation grazing treatments. CK—enclosed steppe; FG—traditional time of grazing; TG—early end of grazing; YG—delayed start of grazing; YT—start late and end early of grazing. Different small letters indicate significant differences among different treatments at *p* < .05.

There were no significant differences in the aboveground biomass of the plant community among treatments (*p* > .05, Figure 3). The aboveground biomass of gramineae in FG, TG, and YG was 62.37%, 63.26%, and 65.69 higher than that in CK (*p* < .05), respectively. There was no significant difference in the aboveground biomass of gramineae under different warming rotation grazing treatments (FG, TG, and YG) (*p* > .05). However, compared with CK, the aboveground biomass of leguminosae in YG and YT decreased by 39.31% and 57.57% (*p* < .05), respectively. Treatments YG and YT had decreases of 31.18% and 48.37% in aboveground biomass of leguminosae in comparison with FG, respectively (*p* < .05). The biomass of forbs in FG and YG significantly decreased by 93.80% and 84.71% than that in CK (*p* < .05). The aboveground biomass of forbs in TG and YT increased by 64.56% and 77.64% compared with FG, respectively. Therefore, traditional time and delaying the start of grazing are conducive to the growth of gramineous plants and can appropriately reduce the proportion of forbs.

### 3.2 Response of the plant species diversity to warming seasonal grazing

**FIGURE 4:**
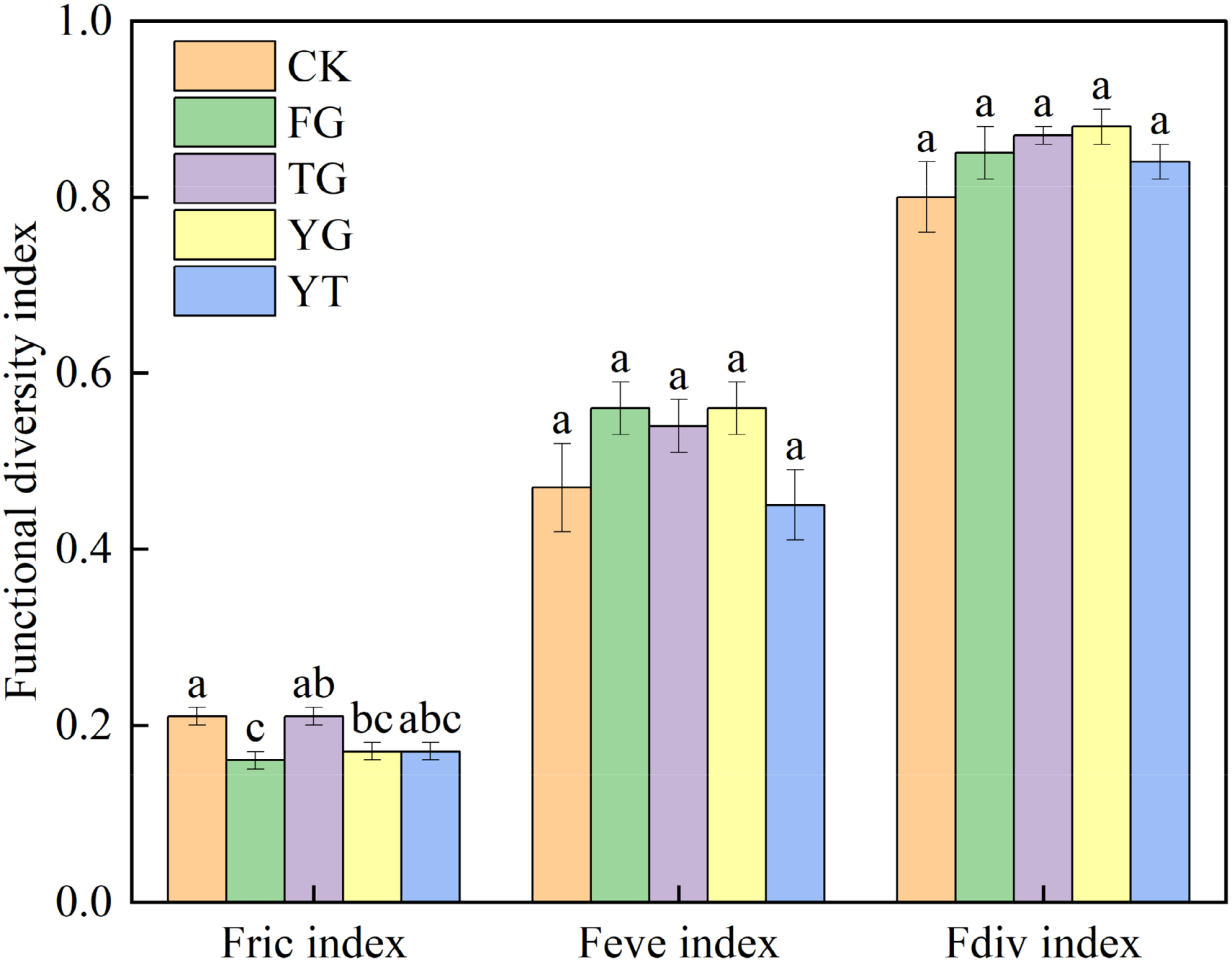
Changes in plant functional diversity index under warming seasonal rotation grazing treatments. CK—enclosed steppe; FG, traditional time of grazing; TG—early end of grazing; YG—delayed start of grazing; YT—start late and end early of grazing. Fric index, functional richness index; Feve index, functional evenness index; Fdiv index, functional divergence index. Different small letters represent significant differences among different treatments at *p* < .05.

Patrick's index in YT (17.70) and CK (17.20) was the largest, followed by TG (16.10), and YG (15.50), and the FG (13.90) was the lowest (Table 2). The Patrick's index in the FG decreased by 21.47% and 19.19% compared with YT and CK (*p* < .05). The YT had an increase of 21.67% and 15.71% compared with FG and YG (*p* < .05). Compared with CK, warming seasonal grazing treatments (FG, TG, YG, and YT) had no significant effect on the Shannon–Wiener index (*p* > .05). However, the Shanon–Wiener index in the YT increased by 21.43% and 15.71% compared with FG and YG (*p* < .05). There were no significant differences in Pielou index and Simpson index among different grazing treatments (*p* > .05). However, the Pielou index and Simpson index in YT increased by 11.11%–15.94% and 3.66%–11.84% compared with CK, FG, TG, and YG, respectively. Therefore, the grazing time treatments had no significant effects on the functional richness and evenness of the plant community. There were no significant differences in Fric index among TG, YT, and CK the same pattern of the Fric index occurred in FG, YG, and YT (*p* > .05, Figure 4). The Fric index in FG and YG significantly decreased by 19.05%–23.81% compared with CK and TG (*p* < .05). Compared with FG, the Fric index in the seasonal rotational grazing treatments (TG, YG, and YT) increased by 6.25%–31.25%. Warming seasonal grazing slightly increased the Fdiv index of the community; however, there was no significant difference between CK and the grazing plots (*p* > .05). Compared with CK, the Fdiv index in the seasonal rotational grazing treatments (FG, TG, YG, and YT) increased by 5.00%–10.00%. Also, no significant difference in Feve index was observed under different warming seasonal grazing treatments (*p* > .05). Compared with CK, the Feve index in the seasonal rotational grazing treatments (FG, TG and YG) increased by 14.89%–19.15%, and YT decreased by 4.26%; Compared with FG, the Feve index in the seasonal rotational grazing treatments (TG, YT) decreased by 3.57%, 19.54%, respectively.

**TABLE 2:** Changes in plant species diversity index warming seasonal rotation grazing treatments

Treatments	Patrick index	Shanon–Weiner index	Pielou index	Simpson index
CK	17.20 ± 0.58a	2.05 ± 0.05ab	0.72 ± 0.02a	0.82 ± 0.01a
FG	13.90 ± 0.60b	1.82 ± 0.08b	0.69 ± 0.02a	0.76 ± 0.02a
TG	16.10 ± 0.77ab	1.96 ± 0.08ab	0.71 ± 0.02a	0.79 ± 0.02a
YG	15.50 ± 0.07ab	1.91 ± 0.17b	0.70 ± 0.05a	0.76 ± 0.06a
YT	17.70 ± 0.87a	2.21 ± 0.08a	0.80 ± 0.02a	0.85 ± 0.01a

*Note*: CK—enclosed steppe; FG—traditional time of grazing; TG—early end of grazing; YG—delayed start of grazing; YT—start late and end early of grazing. Different small letters represent significant differences among different treatments at *p* <0 .05.

### 3.3 Effect of soil properties on plant community diversity

**FIGURE 5:**
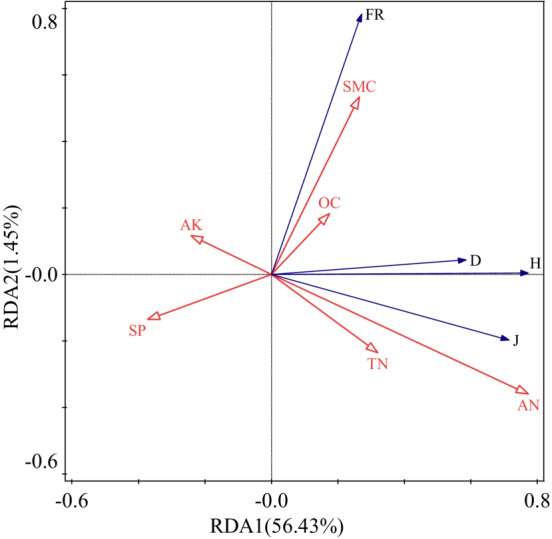
Plant diversity index and RDA ordination map of soil environment factors. AN, SP, SMC, TN, AK, and OC represent available nitrogen, soil porosity, soil moisture content, total nitrogen, available potassium, and organic carbon. H, D, J, FR represent Shannon–Wiener index, Simpson index, Pielou index, and FRic index. The red arrow represents the ecological factors (explanatory variables), the blue arrow represents the plant diversity index (corresponding variables), the cosine value of the angle between the hands represents the correlation between the two, and the length of the explanatory variable needle represents the degree of interpretation of the response variable, and the longer the needle, the stronger the degree of understanding.

The results of redundancy analysis showed that the eigenvalues of the first two sorting axes were 0.5643 and 0.0145, respectively. The first axis explained 56.43% of the relationship between explanatory and response variables, and the second axis explained 1.45%. The correlation coefficients between environmental factors and community diversity in the first two axes were 0.7663 and 0.8484 (Table 3), respectively. Soil properties (TN, AN, OC, and SMC) were positively correlated with J, H, and D, and negatively correlated with AK and SP; the response of soil alkali hydrolyzed nitrogen to plant diversity was significantly explained (*p* < .05). Soil properties (OC, SMC, and AK) were positively correlated with FRic. Furthermore, analysis of the explanatory ability of soil to plant community structure, indicated that soil alkali hydrolyzed nitrogen was the largest factor to explain plant community changes, with an explanatory amount of 34.0% and a contribution rate of 57.92% (Table 4). Therefore, under grazing disturbance in the study area, soil available nitrogen is the dominant factor of plant community species diversity change (Figure 5).

**TABLE 3:** RDA analyses for vegetation community characteristics and soil factors

Parameters	Axis1	Axis2	Axis3	Axis4	Total explained variance (%)
Eigenvalues	0.5643	0.0145	0.0073	0.0010	58.7
Explained fitted variation (Cumulative) (%)	96.12	98.59	99.83	100	
Species‐environment correlations	0.7663	0.8484	0.7947	0.3706	

**TABLE 4:** The correlation coefficients, explanatory quantity, and contribution rate of environment variable and ordination axes of redundancy analysis (RDA)

Environment factors	Explains the amount of variation (%)	Contribution (%)	Pseudo‐F	*p*
AN	34.0	57.92	6.7	.018
SP	12.1	20.61	2.7	.118
SMC	9.3	15.84	2.2	.138
TN	1.6	2.73	0.3	.572
AK	1.1	1.87	0.2	.638
OC	0.6	1.02	0.1	.776

*Note*: AN, SP, SMC, TN, AK, and OC represent available nitrogen, soil porosity, soil moisture content, total nitrogen, available potassium, and organic carbon.

## 4 DISCUSSION

### 4.1 Warming seasonal grazing on vegetation community characteristics

Grazing is the primary disturbance factor of the natural steppe system. It mainly affects the steppe community structure and species composition by regulating the community species and functional diversity (Fedrigo et al., 2017). Among, the animal feeding and trampling directly affect the structure and composition of the steppe plant community and then affect the proportion of plant species and quantity in the community (Kraaij & Milton, 2006). In this study, short‐term warming seasonal had no significant effects on the composition of the dominant plants in desert steppe, which mainly includes *A. mongolicum* and *S. breviflora* of the gramineae, *L. potaninii* of the leguminosae, etc. And this result was similar to the finding of Song et al. (2020) who concluded that grazing starting in June facilitated the maintenance of grassland productivity and stable community structure in the temperate grasslands of Inner Mongolia while not altering their dominant plant composition (*Leymus chinensis*). The main reason is that perennial plants with developed roots increase the competitive advantage of resources compared with primary and biennial herbs (Yu et al., 2021). Therefore, short‐term rotational grazing had no significant effect on dominant species. In different grazing plots, the importance value of gramineae increased significantly, while the significance of leguminosae decreased significantly. A better explanation is that the grazing tolerance of leguminous forages is lower than that of gramineae (Zhang, Dong, et al., 2018), and better palatability of leguminosae (Pulido et al., 2016). Our results showed that enclosed were not conducive to the growth of gramineous grasses, traditional and delaying the start of grazing was more conducive to the growth of gramineous plants. It could appropriately reduce the proportion of weeds, and this result was different from that of Karami et al. (2019), this may be mainly related to the short grazing time. Delayed grazing can provide sufficient time for the growth and development of herbage and promote the fruiting of perennial plants. Furthermore, it offers robust conditions for the protection of pasture grass seed resources and the reproduction of fine pasture grass; reducing the disturbance of livestock to steppe vegetation and the occupation of toxic weeds can also promote the optimization of community structure (Luo et al., 2014).

### 4.2 Warm‐seasonal grazing on vegetation diversity

Species diversity represents a dimension of biodiversity, functional diversity is based on other dimensions of biodiversity, and the combination of species diversity can better explain changes in community composition, structure, and function. Community species diversity plays an essential role in maintaining the stability of ecosystem structure and function in desert steppe (Chao et al., 2014). Animal feeding is one of the critical factors controlling species diversity (Liu et al., 2015). Grazing disturbance leads to changes in plant community stability, plant community structure, and species diversity. The results of this experiment showed that short‐term seasonal grazing mainly affects the species richness index in desert steppe. Due to higher grazing frequency resulting in lower species richness index, reducing the frequency of grazing disturbance was more beneficial to maintain higher species richness. With the livestock feeding frequency increasing, some species in the community disappeared, and the richness index of the community decreased (Dorrough et al., 2007). In our study, the Patrick index of YT is higher than that of CK, indicating that lower grazing frequency is conducive to the improvement of species richness, and the same result was reported by Zhang, Dong, et al. (2018) who considered that with the grazing intensity increasing, the species diversity of the community decreased significantly. The increase in livestock feeding frequency will lead to the disappearance of some species in the community and reduce the richness index of the community. The Shanon–Wiener index of YT (delayed start and early end of grazing) was the highest, this result indicated that low‐frequency grazing in warm season can promote the increase in the Shanon–Wiener index, a similar result was obtained by Karami et al. (2019). In the sample plots with low grazing frequency, livestock feeding weakens the competitiveness of dominant species, provides opportunities for the invasion of some species, and increases the species diversity of the community. The feeding of livestock will also affect the sexual reproduction of plants and change the community structure (Han & Ritchie, 1998). The number of plant flowers reduced firstly and then the seed setting rate decreased after livestock feeding in short growth seasons, resulting in the reduction of the proportion of vegetation sexual. Species richness and diversity index declined with high‐frequency grazing in the growing season, which resulted in the reduction of the proportion of vegetation sexual (Herrero‐Jáuregui & Oesterheld, 2018; Wang, 2000).

Species diversity refers to the species‐specific variability of biodiversity. Functional diversity refers to the overall differences in functional traits among species in the community, controlling by the interaction between species and environmental factors. Functional and species diversity are critical predictors of ecosystem function (Huang et al., 2019). To reveal the relationship between functional diversity and ecosystem function, functional diversity is often defined as functional richness, functional evenness, and functional divergence (Mouchet et al., 2010). Our results showed that the community functional richness index was more sensitive to grazing, and grazing could reduce the community functional richness index in desert steppe. The main reason is that grazing can weaken the competitive advantage of dominant species, change the niche of species in steppe, cause fierce species competition, and reduce the functional richness index (Schultz et al., 2011). Delayed grazing had a significant impact on the later growth of vegetation and reduces the functional richness of the community. In the grazing plots that ended in advance, the functional richness index is high, which means that the niche of each species in the community is fully occupied. The superposition of various characteristics of species increases the richness of functional traits in the community, which can more effectively buffer the interference of the external environment, increase the anti‐interference ability, and the community also has higher stability (Tilman, 1996).

### 4.3 Relationship between plant community diversity and soil environmental factors

Grazing is one of the important reasons that affect grassland diversity. The selective feeding of livestock has changed the functional characteristics of plants, species composition, and diversity of communities (Niu et al., 2015). At the same time, the comprehensive effects of livestock trampling, excretion, and other activities will also change the soil properties (Deng et al., 2017). Soil is the carrier for plants to survive. Its properties not only affect the distribution of plants but also affect the diversity of plant communities (Wan et al., 2018). Audet et al. (2015) found that the species richness of plants in the riparian wetlands of Nordic Denmark is mainly affected by soil phosphorus content. The more competitive species are dominated in the community under higher soil nutrients, which can limit the growth of other species and ultimately reduce plant community diversity. Ding et al. (2018) determined that total nitrogen and bulk density in soil factors are the main soil factors affecting plant diversity through Pearson correlation analysis, stepwise multiple linear regression, and RDA. In this study, RDA analysis showed that available nitrogen in environmental factors was positively correlated with the Shannon–Wiener, Simpson index, and Pielou index. Our results agree with those observed by Ning et al. (2019) who reported that there was a positive correlation between soil nitrogen and species richness, Shannon–Wiener, Simpson index in sandy grassland. A better explanation is that nitrogen is the primary limiting resource of plant productivity and the main factor affecting the growth of grassland plants (Li et al., 2010). In the study, YG has the highest available nitrogen content (*p* > .05) (Table 5), which shows that delayed rotational grazing in the warming season mainly affects the nitrogen absorption of plants by affecting the important value and functional group composition of plants, thus affecting plant species diversity, which may be related to the accumulation of excreta or litter of grazing livestock (Zhou et al., 2017).

**TABLE 5:** Effects of rotational grazing in different warming seasons on soil physical and chemical properties

Treatments	AN (mg·kg^−1^)	SP (%)	SMC (%)	TN (g·kg^−1^)	AK (mg·kg^−1^)	OC (g·kg^−1^)
CK	5.35 ± 0.46ab	51.86 ± 2.04b	13.01 ± 2.63a	0.30 ± 0.03a	53.50 ± 3.57ab	8.37 ± 0.75a
FG	4.94 ± 0.38ab	53.12 ± 0.29ab	10.55 ± 0.21ab	0.26 ± 0.01ab	65.07 ± 6.11a	6.72 ± 0.24ab
TG	4.71 ± 0.25b	52.51 ± 2.31b	7.34 ± 0.90b	0.24 ± 0.01b	66.04 ± 0.27a	5.99 ± 0.37b
YG	5.99 ± 0.27a	58.08 ± 1.05a	10.11 ± 0.39ab	0.28 ± 0.02ab	47.59 ± 4.94b	6.85 ± 0.44ab
YT	5.75 ± 0.35ab	56.03 ± 0.37a	9.65 ± 0.25ab	0.26 ± 0.01ab	53.10 ± 3.65ab	6.32 ± 0.85b

*Note*: CK—enclosed steppe; FG—traditional time of grazing; TG—early end of grazing; YG—delayed start of grazing; YT—start late and end early of grazing. AN, SP, SMC, TN, AK, and OC represent available nitrogen, soil porosity, soil moisture content, total nitrogen, available potassium, and organic carbon. a, ab, b indicated significant differences among different treatments at p < .05.

## 5 CONCLUSION

Short‐term warming seasonal rotational grazing had no effect on the composition of the dominant plants in gramineae and leguminosae in desert steppe. Delayed grazing provides sufficient time for the growth and development of herbage and promotes the growth of perennial plants, which can benefit the growth of gramineous plants, optimize the composition of the community structure, and control weed growth. Traditional grazing reduced the community evenness (Patrick index) and functional richness index, while the delayed start and early end of grazing can promote community evenness and species diversity (Shannon–Wiener index). Thus, the delayed start of grazing is recommended in warming seasons. These findings propose effective management measures to improve important value and biomass of gramineous plants, and maintain vegetation diversity and sustainability in desert steppe.

## AUTHOR CONTRIBUTIONS

**Wen Li:** Data curation (lead); formal analysis (lead); methodology (lead); project administration (lead); software (lead); writing – original draft (lead); writing – review and editing (lead). **Tingting Su:** Investigation (equal); methodology (equal); resources (supporting). **Yan Shen:** Funding acquisition (equal). **Hongbin Ma:** Funding acquisition (equal); methodology (equal); resources (equal). **Yao Zhou:** Methodology (equal). **Qi Lu:** Investigation (equal); methodology (equal). **Guohui Wang:** Resources (equal); writing – review and editing (equal). **Zhuo Liu:** Methodology (equal). **Jianping Li:** Methodology (equal).

## CONFLICT OF INTEREST

The authors declare that they have no known competing financial interests or personal relationships that could have appeared to influence the work reported in this paper.

## Data Availability

The data that support the findings of this study are openly available in Figshare at https://doi.org/10.6084/m9.figshare.20278296.v1.
